# Placenta-Specific miR-125b Overexpression Leads to Increased Rates of Pregnancy Loss in Mice

**DOI:** 10.3390/ijms23020943

**Published:** 2022-01-15

**Authors:** Fen Sun, Hui Cai, Lunbo Tan, Dezhe Qin, Jian Zhang, Jinlian Hua, Xiujun Fan, Sha Peng

**Affiliations:** 1 Shaanxi Centre of Stem Cells Engineering & Technology, College of Veterinary Medicine, Northwest A&F University, Yangling, Xianyang 712100, China; fensun2017@126.com (F.S.); 18346700148@163.com (H.C.); dezheqin@163.com (D.Q.); jinlianhua@nwsuaf.edu.cn (J.H.); 2 Laboratory of Reproductive Health, Shenzhen Institute of Advanced Technology, Chinese Academy of Sciences, Shenzhen 518055, China; lunbo.tan@outlook.com; 3 Center for Reproduction and Health Development, Institute of Biomedicine and Biotechnology, Shenzhen Institute of Advanced Technology, Chinese Academy of Sciences, Shenzhen 518055, China; jian.zhang@siat.ac.cn

**Keywords:** pregnancy loss, angiogenesis, HTR8 cells, JEG3 cells, *VEGF*

## Abstract

Pregnancy loss (PL) is one of the common complications that women can experience during pregnancy, with an occurrence rate of 1 to 5%. The potential causes of pregnancy loss are unclear, with no effective treatment modalities being available. It has been previously reported that the level of miR-125b was significantly increased in placentas of PL patients. However, the role of miR-125b in the development of PL still remains unknown. In the current study, an miR-125b placenta-specific over-expression model was constructed by lentiviral transfecting zona-free mouse embryos followed by embryo transfer. On gestation day 15, it was observed that the placenta was significantly smaller in the miR-125b placenta-specific overexpression group than the control group. Additionally, the abortion rate of the miR-125b placenta-specific overexpression group was markedly higher than in the control group. The blood vessel diameter was larger in the miR-125b-overexpressing specific placenta. In addition, miR-125b-overexpressing HTR8 and JEG3 cell lines were also generated to analyze the migration and invasion ability of trophoblasts. The results showed that miR-125b overexpression significantly suppressed the migration and invasion ability of HTR8 and JEG3 cells. Overall, our results demonstrated that miR-125b can affect embryo implantation through modulating placenta angiogenesis and trophoblast cell invasion capacity that can lead to PL.

## 1. Introduction

Successful reproduction is both biologically and epidemiologically complex, while the loss of a pregnancy following conception is a commonly observed event. An accurate identification of PL in the early stages of gestation is very difficult [1]. PL is the most common pregnancy-associated complication. It is defined as the two or more continuous pregnancy losses in the first or early second trimester of gestation. It occurs in between 0.8% and 1.4% of couples [2]. The etiology of PL is rather complex, and it remains difficult to treat. The various causes of spontaneous abortion in females have been generally identified, including environmental factors, genetics, endocrine, placental anomalies, hormonal problems, infections and psychological trauma [3]. However, in approximately half of the patients with spontaneous abortion, the causes are still unclear.

MiRNAs account for about 1% of predicted genes in animal and human genomes. It has been reported that approximately 30% of these genes may be regulated by miRNAs [4]. A number of studies have demonstrated that miRNA expression in human placenta is abundant and tissue-specific [5,6]. Among the most important miRNA families, the miR-125 family has been reported to be implicated in a variety of diseases. These include various cancers, such as bladder cancer, breast cancer and squamous cell carcinoma [7,8,9]. Accumulating evidence from previous studies also demonstrates that miR-125b could significantly inhibit cell proliferation, migration, invasion and angiogenesis of hepatocellular cancer cells, and its overexpression results in a worse patient prognosis of oral squamous cell carcinoma [10]. MiR-125b includes miR-125b-1 and miR-125b-2. MiR-125b is widely distributed from nematodes to humans. MiR-125b plays an important role in the regulation of different cellular processes such as cell differentiation, proliferation and apoptosis [11]. A few studies have shown miR-125b to be closely related to PL. For instance, one study has demonstrated that expression of hsa-miR-125b-2 in the villi of PL patient was significantly increased [12]. Hsa-miR-125b-2, hsa-miR-520f, hsa-miR-3175, and hsa-miR-4672 can also regulate the expression of the various target genes associated with immune and angiogenesis, leading to PL [13,14]. Additionally, another study has indicated that environmental factors such as smoking can also lead to PL. Meanwhile, miR-125b expression has been found to be closely associated with smoking, as observed in multiple air pollution studies [15]. It may also be related to PL development. The human endometrium undergoes morphological and biochemical modifications right before embryo implantation. The expression of miR-125 has been demonstrated in the human endometrium from the early to mid-luteal phase and was observed to be increased in the ovaries of endometriosis patients [16,17]. Moreover, the change in miR-125b-5p expression in the placenta has been associated with the reduced Doppler of cerebral hemodynamics [18]. However, it is still not clear that how miR-125b can affect the development of placenta.

MiR-125b may regulate many target genes involved in the development of different diseases. *Vascular endothelial growth factor (VEGF)* is one of the most important target genes that can regulate the process of angiogenesis. A large number of studies have reported the potential relationship between PL and *VEGF*. For example, *VEGF* can effectively influence trophoblast cell proliferation and invasion, embryo implantation and promote placenta angiogenesis and the growth of maternal and fetal blood vessels in the uterus [19,20]. There are also reports suggesting that *VEGF* mRNA levels in the chorionic villus tissue of women with abortion were significantly higher than in the control group and could play an important role in fetal and placental angiogenesis [21,22,23]. It has also been demonstrated that *VEGF* can play a vital role in promoting angiogenesis and affecting trophoblast cell proliferation that can lead to abortion [24,25]. Additionally, some studies have also investigated how *VEGF* could potentially affect the pathogenesis of PL patients by regulating the expression of its receptors *VEGFR1* and *VEGFR2*, and then modulating the development of PL [26]. In addition, *VEGF* can be utilized as a potential biomarker for PL risk detection and prevention [27]. At present, it is not clear whether miR-125b can also induce pregnancy loss by regulating the expression of *VEGF*.

MiR-125b has been closely related to PL [12]. Although many companies have developed novel techniques for cancer treatment, such as chemical modifications of miRNA oligonucleotides, an adequate treatment for PL has not been established yet. Furthermore, the specific mechanism of action of miRNA-125b in PL is still unknown and its etiology as well as pathogenesis are still not entirely clear. There also exists a lack of effective treatment and drugs for the management of PL. Thus, it remains an urgent topic for in-depth investigation.

## 2. Results

### 2.1. The Expression of miR-125b Was Upregulated in Samples from PL Patients

**Figure 1:**
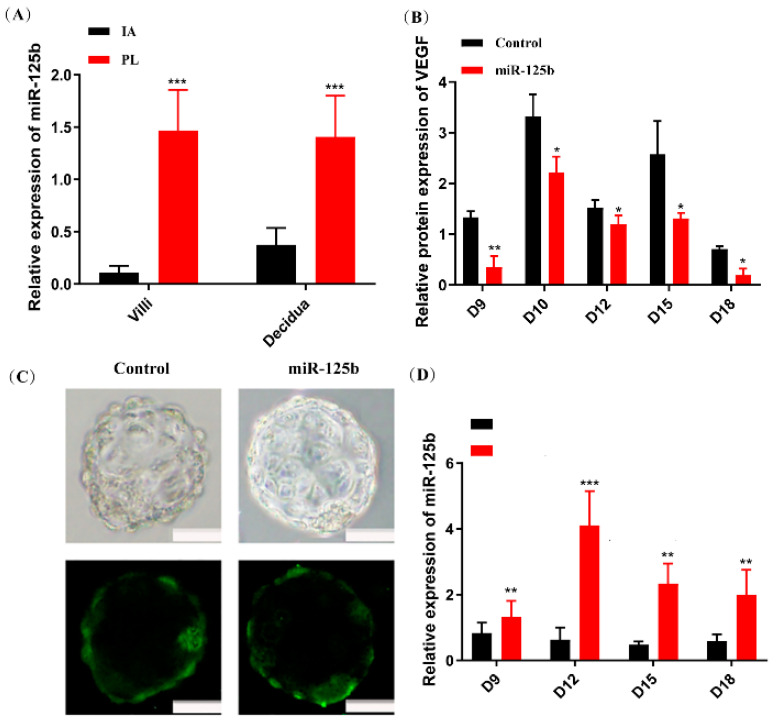
An effective miR-125b expression in PL placentas and mouse model placenta caused by trophoblast-specific miR-125b overexpression. (**A**): The expression of miR-125b in the decidua and villi of 10 PL and IA patients by RT-qPCR. Data were presented as mean ± SEM (*** *p* < 0.01, vs. IA); (**B**): the protein expression of *VEGF* in placenta on different pregnancy days in mice by ELISA. Data were presented as mean ± SEM, *n* = 10 (* *p* < 0.05, ** *p* < 0.01, vs. control); (**C**): Blastocysts transduced with lentiviruses expressing GFP control and miR-125b; Scale bars: 20 μm; (**D**): the expression of miR-125b in placenta of different pregnancy days mice by RT-qPCR. Data were presented as mean ± SEM, *n* = 10 (** *p* < 0.01, *** *p* < 0.001).

The expression levels of miR-125b were examined on the villus and decidua from PL patients and induced abortion (IA) patients. MiR-125b expression was significantly increased in both the villus and decidua of PL patients as compared to IA patients by RT-qPCR (Figure 1A). In contrast, *VEGF* expression was significantly decreased both in the villus and decidua of PL patients (Figure S1).

### 2.2. Placenta-Specific miR-125b Overexpression in a Mouse Model

To further determine the possible role of miR-125b in PL, a lentivirus-based trophoblast-specific transgene expression tool was used [28], which caused an overexpression of miR-125b in placenta trophoblast (Figure 1C). However, through blastocyst transfer, miR-125b levels were observed to be increased as expected in placenta (Figure 1D) and *VEGF* protein level was substantially decreased in placentas of the miR-125b group (Figure 1B).

### 2.3. Placenta-Specific miR-125b Overexpression Affected the Growth of Fetus and Placenta in Mouse

**Figure 2:**
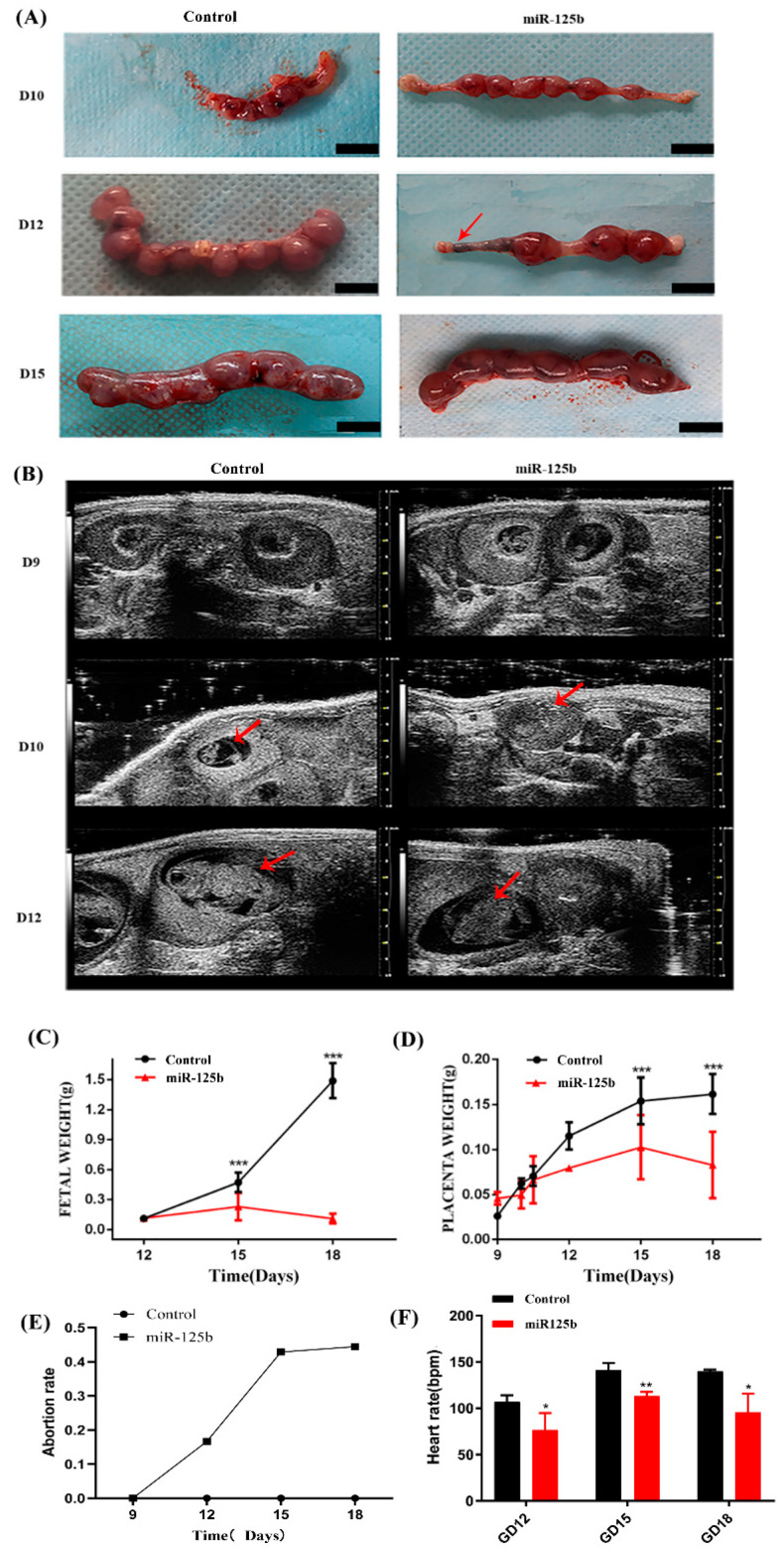
The effect of miR-125b on placenta and fetus in mice (**A**): The placenta and fetus of the GFP group and the miR-125b group on day 10, 12, and 15; (**B**): An ultrasound of mouse on day 9, 10, and 12, the arrows showing embryos; (**C**): the weight of the fetus on different pregnancy days in mice; (**D**): the weight of placenta on the different pregnancy days in mice (*** *p* < 0.001); (**E**): Abortion rate of miR-125b group; (**F**): The heart rate of the mice model on day 12, 15, and 18. Data are presented as mean ± SEM, *n* = 10 (* *p* < 0.05, ** *p* < 0.01).

**Figure 3:**
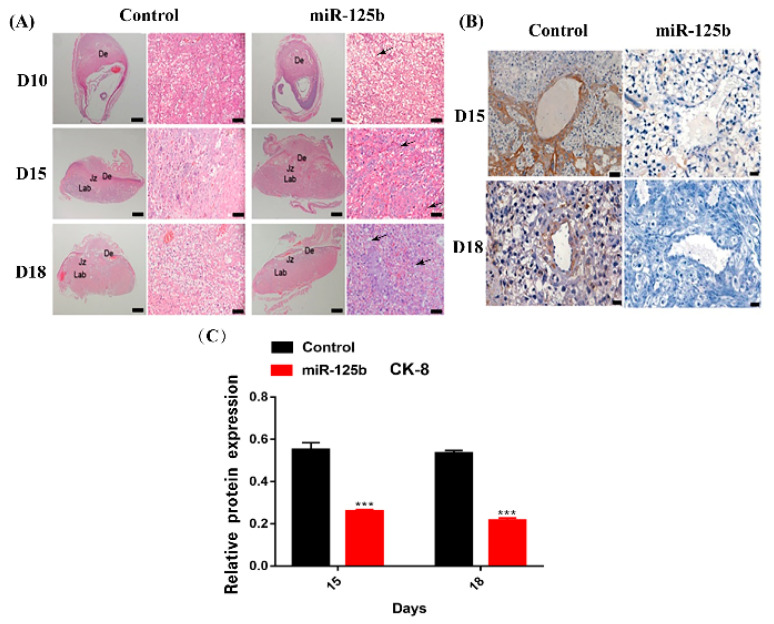
HE and CK-8 immunohistochemistry (IHC) staining of placenta (**A**): HE staining of the placenta of miR-125b group and control group on day 10, 15, and 18, the arrows showing the labyrinth; (**B**): CK-8 IHC staining of the placenta miR-125b group and control group on day 15 and 18 (scale bars: a: 500 μm, 50 μm; b: 20 μm). (**C**): the quantification of CK-8. Data are presented as mean ± SEM, *n* = 3, *** *p* < 0.001 vs. control.

**Figure 4:**
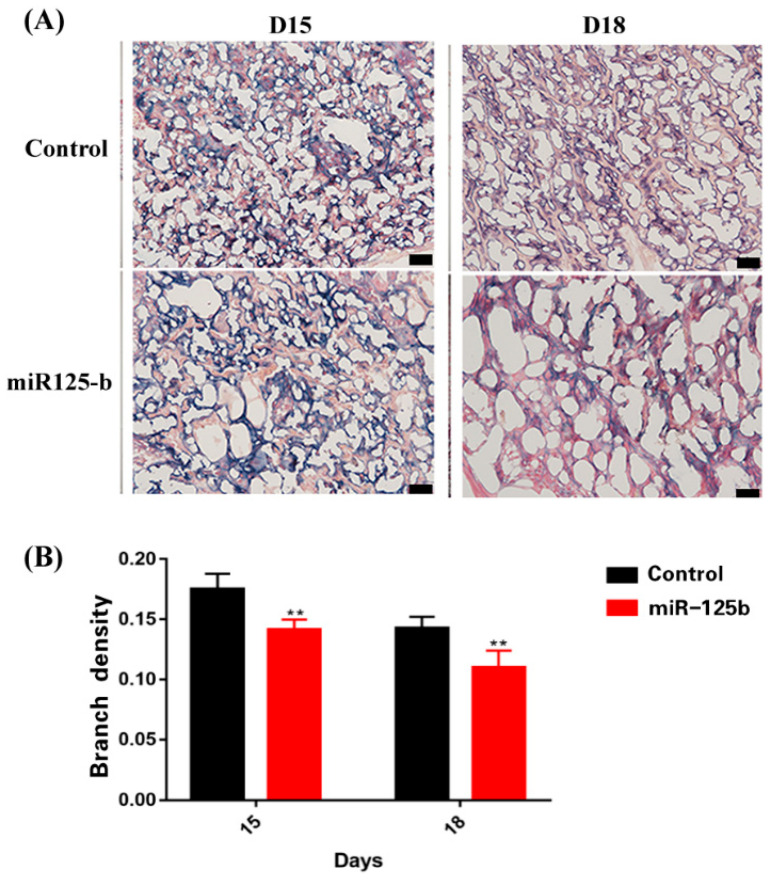
AP staining of placenta (**A**): AP staining of placenta on day 15 and 18. Scale bars: 500 μm and 50 μm; (**B**): Statistical analysis of the branch density compared with the control. Data are presented as mean ± SEM, *n* = 3, ** *p* < 0.01 vs. control.

Among the miR-125bplacenta-overexpression mice, the resorption site was observed on D10, 12, and 15 (Figure 2A); however, only few embryos existed at the implantation site (Figure 2A). On D12, the miR-125b-placenta-overexpression mice only exhibited three implantation sites and the arrows show the fetal reabsorption (Figure 2A). As the result of type-B ultrasonic was analyzed, it was found that there were no significant differences in placentas between miR-125b-placenta-overexpression mice and the control mice on D9 (Figure 2B). However, placenta was not observed in the resorption site of some embryos on D10 and 12, which may indicate that placenta development was possibly restrained (Figure 2B). On D15, only a few normal embryos were observed as the arrows indicate in Figure 2B. Overall, the placental and fetal weights were significantly lower in miR-125b-placenta-overexpression mice as compared to the control groups on D12, 15, 18 (*p* < 0.05) (Figure 2C,D). It was observed that the average fetal weight of the miR-125b-placenta-overexpression mice was under 0.3 g, while the average placental weight of the control mice was up to 1.5 g (Figure 2C). The average placental weight of the miR-125b-placenta-overexpression mice was under 0.1 g, while the average placental weight of the control mice was up to 0.15 g (Figure 2D). Similarly, the heart rate was significantly decreased on D12, 15, and 18 as compared to the control group (Figure 2F). Moreover, abortion rate (vs. D9) was significantly higher on D12, 15, and 18 (Figure 2E). On D12, the abortion rate (embryos vs. embryos of D9) was lower at 0.2, while on D15, the abortion rate was up to 0.5 (vs. D9) (Figure 2E). Histological examination revealed significant changes in the morphologies and mean diameters of the trophoblast-lined spiral arteries (SpAs). In the labyrinth, there were more nucleated cells; thrombus and vacuolation was also noted as indicated with arrows (Figure 3A–C). There were also significant changes in the depth of trophoblast invasion into the decidua or SpAs, as determined by CK8 immunohistochemistry (Figure 3B). On D15 and 18, there was less CK8 colored around the blood vessels (Figure 3B). Moreover, the branch density of the blood vessels was significantly much less quantified using ImageJ software (National Institutes of Health) (Figure 4B) on D15 and D18 compared to the control group. Thereafter, the protein level of *VEGF* was detected in the placenta by ELISA and the expression of *VEGF* was found to be significantly inhibited (Figure 1B). In order to further explore whether miR-125b was associated with modulation of *VEGFA* activity, a dual-luciferase reporter assay in 293T cells was performed. It was found that compared with mimic NC-transfected 293T cells, the luciferase activity of wild-type *VEGFA* following transfection with the miR-125b mimics was markedly reduced (Supplementary Figure S2A,B). Therefore, we concluded that miR-125b might affect the blood vessels by inhibiting the expression of *VEGF*.

### 2.4. MiR-125b Overexpression Affected the Growth of HUVECs

When HUVECs were treated with miR-125b mimics, the cell viability of HUVECs was found to be markedly inhibited as observed by colony forming assay (Supplementary Figure S3A,C). Tube formation assay was used to determine whether miR-125b has antiangiogenic effects on HUVECs. In the control group, the HUVECs cultured showed a tube-like structure or a tube network form, and miR-125b mimics impaired the tube formation of these cells (Figure S3B). MiR-125b significantly inhibited the branch density (Figure S3D) of HUVECs compared with the control group. Then, we examined the possible effects of miR-125b on the cell invasion and migration of HUVECs. As shown in Supplementary Figure S3, the migration (Figure S4A,B) and invasion potential (Figure S4A,C) of HUVECs was significantly suppressed when HUVECs were treated with miR-125b mimics. Epithelial–mesenchymal transition (EMT) has been closely related to cell invasion and migration. Next, we examined whether miR-125b can inhibit the invasion and migration abilities of HUVECs by inhibiting EMT. We performed Western blotting to examine the protein expression levels of N-cadherin, E-cadherin, vimentin and Snail related to the EMT pathway. It was observed that miR-125b significantly reduced the protein expression levels of N-cadherin (Figure S4D,F), Snail (Figure S4D,G) and vimentin (Figure S4D,H). Moreover, miR-125b significantly increased the protein expression levels of E-cadherin (Figure S4D,E). Therefore, the inhibition of the EMT pathway may result in reduced invasion and migration of HUVECs.

### 2.5. Overexpression of miR-125b Can Alter Cell Migration and Invasion by Regulating the Expression of VEGF

**Figure 5:**
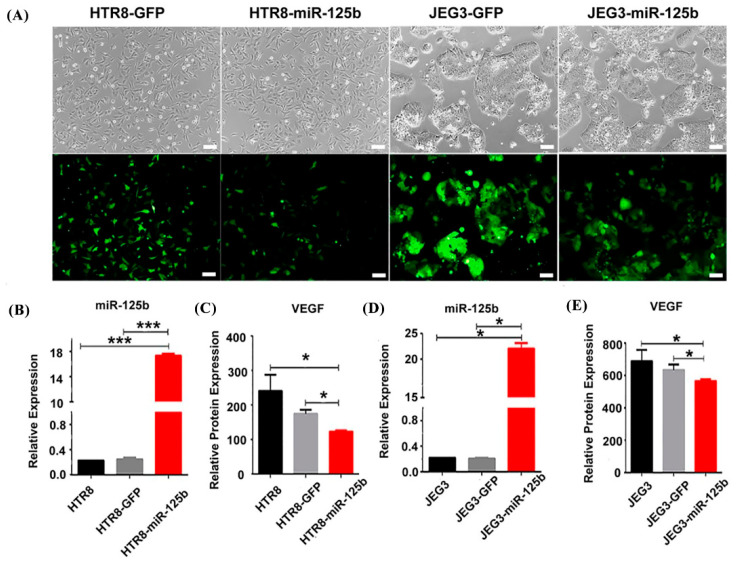
Establishment of HTR8 and JEG3 cell lines that can express miR-125b and regulate the expression of *VEGF* (**A**): HTR8 and JEG3 cell lines that express GFP (HTR8-GFP, JEG3-GFP) and miR125b (HTR8-miR125b, JEG3-miR125b); images as observed under the microscope 10 times; (**B**) and (**D**): the expression of miR-125b in HTR8 and JEG3 cell lines by RT-qPCR. Data are presented as mean ± SEM, *n* = 3 (* *p* < 0.05; *** *p* < 0.01); (**C**,**E**): the protein expression of *VEGF* in HTR8 and JEG3 cell lines by ELISA. Data were presented as mean ± SEM, *n* = 3 (* *p* < 0.05).

**Figure 6:**
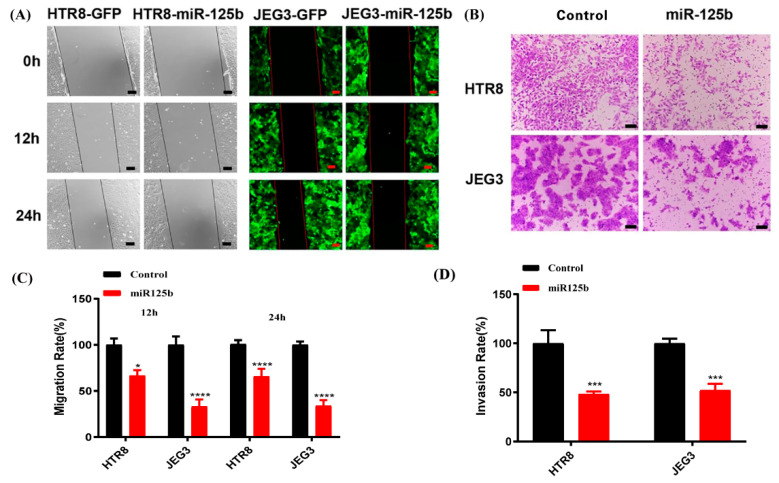
The effect on the migration and invasion ability of HTR8 and JEG3 cell lines after miR-125b treatment. (**A**): The effect on the migration ability of HTR8 and JEG3 cell lines after miR125b treatment; (**B**): the effect on invasion ability of HTR8 and JEG3 after miR-125b treatment; (**C**,**D**): data are presented as mean ± SEM, *n* = 3. * *p* < 0.05, *** *p* < 0.01, **** *p* < 0.001.

To examine the possible effects of miR-125b in placenta, miR-125b was overexpressed in both JEG3 and HTR8 trophoblast cell lines by RT-qPCR (Figure 5A). We observed results similar to the in vivo results in miR-125b-expressing cells, and the VEGF level was significantly decreased by ELISA (Figure 5B). At the same time, the migration and invasion potential of HTR8 and JEG3 cells was also significantly suppressed when miR-125b was overexpressed (Figure 6A–D) (*p* < 0.05). Quantification analysis also indicated that the invasiveness of HTR8 and JEG3 cell lines was reduced by 70% and 50% (*p* < 0.05) (Figure 6C), whereas the migration of HTR8 and JEG3 cell lines was reduced by 60% and 50% (*p* < 0.05) (Figure 6D).

## 3. Discussion

PL etiology tends to be complex, and a number of conditions can increase the potential risk of PL. In this study, the embryo transfer technique was used to potentially assess important regulatory relationship between increased placental miR-125b and decreased *VEGF* in PL. Placenta formation and development are key in the maintenance of optimal pregnancy and fetal growth as it can facilitate an effective nutrition and substance exchange between the mother and the fetus. The labyrinth is a highly vascular part of the placenta, which controls the elimination of the waste gases and allows an optimal nutrient circulation between the mother and the fetus [29]. Extensive angiogenesis is a critical process for pregnancy establishment. Moreover, any labyrinth vessel anomalies can potentially lead to the placental perfusion, oxygen, and nutrient transport to be impeded [30]. It has been reported that some genetic mutations can also result in the developed labyrinth and reduce the vascular density, thus causing fetal death [31]. Meanwhile, *VEGF* plays a crucial role in regulating the angiogenesis and vasculogenesis of human pregnancy from early to later stages [32]. It can be utilized as a possible biomarker for PL risk detection and prevention [32,33]. The protein level of *VEGF* was significantly decreased in the placentas of miR-125b mice. Mice lacking *VEGF* expression died in utero due to inadequate vascular formation [34]. However, there is unreported evidence to suggest that miR-125b might have an important effect on PL to mediate *VEGF* expression. A number of previous studies have indicated that miR-125b plays a critical role by damaging the placenta and fetus. The findings have also suggested that the weight of the placenta and fetus was lower and placental blood vessels were decreased. Moreover, the results also indicated that miR-125b expression was significantly increased and *VEGF* expression was decreased in PL patients compared to IA patients analyzed with RT-PCR (Figure S3). The findings of the dual-luciferase reporter assay showed that *VEGF* was one of the target genes of miR-125b. The observations also demonstrated that *VEGF* expression in the placenta was significantly decreased after an overexpression of miR-125b in the mice. Overall, our results showed that miR-125b inhibited placental angiogenesis by downregulating *VEGF* levels. This result can lead to potential critical implications for designing PL therapies that could effectively manipulate miR-125b levels, such as miR-125b inhibitor.

One of the most important placenta cells is trophoblasts, which play an important role in embryo implantation and the decidua of a uterus. During early embryonic differentiation, the trophoblast cells derived from blastocyst ectodermules develop into a placenta. An embryo relies on identification, adhesion and invasion of the trophoblast cells in the endometrium for proper implantation into the uterus [29]. Trophoblast cells can secrete a variety of different hormones and cytokines for maintaining normal embryo development, thereby facilitating maternal–fetal nutrition and substance exchange. At the end of pregnancy, the trophoblast cells can be spurred to be eliminated from the uterus by producing different proteases [29]. Placental and organ formation is the key for maintaining proper fetus growth and development [35]. However, the invasion of endometrial villous trophoblast cells and syncytio trophoblast formation primarily control the placental function [36]. A few studies have indicated that miR-125b could inhibit the trophoblast cell invasion by regulating S1PR1 (sphingosine-1-phosphate receptor 1) expression [35]. In addition, others have reported that EG-VEGF (endocrine gland-derived vascular endothelial growth factor) could activate ERK1/2 (extracellular regulated protein kinases) signaling and the subsequent up-regulation of MMP2 (matrix metalloproteinase 2) and MMP9 (matrix metalloproteinase 9), thus promoting cell invasion in human trophoblast HTR-8/SVneo cells [37,38]. In this study, miR-125b significantly suppressed the migration and invasion ability in HTR8 and JEG3 cells, which may affect the formation of the blood vessels. Furthermore, CK-8 is expressed homogeneously in placental and intravascular trophoblast cells [39]. Here, positive CK-8 staining in the placenta of miR-125b model mice was observed to be significantly weaker than in the control group, especially around the spiral arteries. These results suggested that miR-125b might cause structural abnormalities in the placental vasculature by inhibiting the trophoblast cell infiltration. MiR-125b can affect the trophoblast cells’ function of supporting and regulating the development and growth of the placenta and fetus. Nevertheless, providing an embryo with nutrition and then causing fetal growth to suffocate or die might result in pregnancy loss, and is not considered as normal.

In conclusion, an association of miR-125b with miscarriage has been demonstrated. VEGF can serve as an important target gene of miR-125b that has been closely related to placental angiogenesis and trophoblast cell invasion [26]. This study also indicated a possible relationship between VEGF and miscarriage. The findings indicated that miR-125b could significantly affect placenta angiogenesis by inhibiting VEGF expression, and thus could possibly lead to the development of novel treatment modality for PL.

## 4. Materials and Methods

### 4.1. Patients and Samples

All participants in this study were Chinese. The decidual tissues and villus tissues were obtained from 15 clinically normal pregnancies (NP) (*n* = 10) and PL patients (*n* = 10) that were detected by ultrasound (average age: 28.6 years, average gestational age at sampling 46 weeks). The decidual and villus tissues were stored in TRIzol at −80 °C until further analyses.

### 4.2. Production and Purification of Lentiviral Vector

We constructed three different viral vectors (LV-CMV-has-pri-miR-125b-EF1-copGFP (miR-125b) vector, LV-CMV-mmu-pri-miR-125b-EF1-copGFP vector and LV-CMV-chr9-EF1-copGFP vector). LV-CMV-chr9-EF1-copGFP (GFP) vector served as the control group, which could express any random sequence. The primers used are listed in Table 1. These vectors were produced and purified according to the protocol reported previously [40].

### 4.3. Mouse Model and In Vivo Study Protocol

Female and male CD-1 mice (6–8 weeks old) were purchased from the Medical Experimental Animal Center of Guangdong Province and kept under 12 h light and 12 h dark cycles with free access to food and water. The care of laboratory animals and experiments on animals were carried out in accordance with the protocol approved by the Animal Ethics Committee of the Shenzhen Institutes of Advanced Technology, Chinese Academy of Sciences (SIAT), China (SIAT-IRB-170406-YYS-FXJ-A0350) for Animal Care and Use.

The blastocysts were collected and transduced with LV-CMV-mmu-pri-miR-125b-EF1-copGFP vector and LV-CMV-chr9-EF1-copGFP vectors as described below. The blastocysts were collected and flushed with EmbryoMax M2 Medium (Millipore) on day 4 of pregnancy/pseudo pregnancy (GD4) on 9:00–10:00 AM. Then, the blastocysts were washed in microdrops containing KSOM Embryo Culture media (Millipore) and treated with acid Tyrode’s solution (Sigma, Saint Louis, CA, USA) for removing zona pellucidae. The blastocysts were washed in KSOM and incubated in 5 uL KSOM drops containing 2.5 × 10^9^ particles/mL concentrations of virus under light mineral oil (Irvine Scientific, Santa Ana, CA, USA) for 6 h. The transduced blastocysts were washed with EmbryoMax M2 medium to remove extra viruses and thereafter transferred into GD3 (on the third day of gestation) pseudo-pregnant mice.

### 4.4. Real-Time PCR

The total RNAs were obtained from the placentas of PL patients, induced abortion pregnant patients, mouse models, as well as HTR8 and JEG3 cell lines. The stem–loop reverse transcription (RT) miRNA quantification method, followed by SYBR green real-time PCR analysis, was used to detect the expression of miRNA. The primers used are listed in Table 1. Real-time PCR was performed using an SYBR RT-PCR kit (Takara, Shiga, Japan) in the iQ5 Detection System (BioRad, Hercules, CA, USA). The internal control in the present study was U6 small-nuclear RNA. The relative expression levels of various miRNAs were analyzed with the ΔΔCT method.

### 4.5. Immunocytochemistry (IHC) of CK-8

Immunohistochemistry of testis was performed on 10 μm sections of paraffin-embedded tissues with a peroxidase labeling kit from Vector Laboratories, by using mouse monoclonal antibody against CK-8 (sc-32328, Santa Cruz, CA, USA). Then, a DAB substrate kit (Vector Laboratories, Burlingame, CA, USA) was used to make the staining visible for peroxidase and counterstained with hematoxylin. The stained sections were then observed under a microscope (Leica, Germany).

### 4.6. Cell Culture and Transfection

The human trophoblast cell line (HTR-8) was a gift from Dr Charles H. Graham, Queen’s University, Canada [41], and the JEG3 cell line was purchased from the Cell Bank of Shanghai, China. These cells were cultured in (D-MEM)/F-12 medium containing 10% fetal bovine serum at 37 °C in 95% air and 5% CO_2_. After two passages, the cells were cultured in a 24-well plate and then transfected with the different lentiviral vectors. After six hours, the cells were cultured in (D-MEM)/F-12 medium with 10% fetal bovine serum for 48h, and the positive cells were selected using the flow cytometry method (4A BIOTECH, Beijing, China).

### 4.7. AP Staining

The frozen sections were washed with 1 × PBS three times, treated with 4% paraform-aldehyde for 10 min, washed with 1 × PBS twice, followed by staining with Alkaline Phosphatase Kit (Millipore, Burlington, MA, USA) according to the manufacturer’s instructions. Thereafter, images were taken by a Leica DMi8 microscope (Leica, Germany) and analyzed using Image Pro Plus 6.0 software.

### 4.8. ELISA

Enzyme-linked immunosorbent assay (ELISA) was used to analyze the level of *VEGF* in the HTR-8 and JEG3 cells and placental supernatant. The cell supernatant was collected after the cell were cultured for 24 h. The placental supernatant was collected after mice were killed on D9, D12, D15, and D18. According to the 0.5 g placenta tissue obtained, 1 mL normal saline was homogenized in a 10 mL EP tube on ice and then centrifuged to collect the supernatant. A commercial ELISA kit (eBiocience, San Diego, CA, USA) was used according to the manufacturer’s instructions for conducting this assay.

### 4.9. The Type-B ultrasonic Assay

The LOGIQ400 ultrasonic diagnostic instrument produced by GE Company in the United States was used, and the probe frequency was 3.5 MHz. The mouse was placed in the supine position and then the fetal structure was examined by routine ultrasound and the fetal heart rhythm was observed.

### 4.10. Cell Migration and Invasion Assays

To analyze the cell migration, a wound healing assay was performed. The cells were cultured in a six-well plate, and scratches were made on the single cell layer with a sterile 20 μL plastic pipette tip. The cells were incubated in a chamber with 95% air and 5% CO_2_ at 37 °C. After 12 h, 24 h, and 48 h, the pictures were captured and the distances were recorded using Image J.

To analyze the invasive ability of trophoblasts, the cells were starved for 24 h in the serum-free media and washed twice with PBS. Then, the cells were resuspended in the serum-free media. The experiment was conducted with the utilization of 24-well plates containing 8 μm cell culture inserts, and every insert contained 1 × 10^5^ cells in a 300 μL suspension. At the bottom of the insert, 700 μL (D-MEM)/F-12 with 10% fetal bovine serum was added as the chemotactic agent and then incubated at 37 °C in 95% air and 5% CO_2_ for 48 h. The insert was fixed with methanol for 30 min and thereafter stained with 0.05% gentian violet for 10 min. The stained cells were counted with Image J and five randomly selected fields were used for the statical analysis.

All the data have been expressed as mean ± SEM throughout, and the statistical significance was assessed by either one-way ANOVA followed by Fisher’s least significant difference test for post hoc comparisons or the Student’s *t*-test (PRISM, GraphPad). *p* < 0.05 was considered as statistically significant.

### 4.11. Western Blot

The cells were lysed using the PRO-PREPTM Protein Extraction solution (iNtRon Biotech). Total proteins were quantified by BCA detection kit (Donghuan Biotech, Shanghai, Japan). The equal amounts of protein were separated by 10% sodium dodecyl sulfate-polyacrylamide gel electrophoresis and transferred to a polyvinylidene difluoride membrane (PVDF) (EMD Millipore Corp., Billerica, MA, USA) and blocked with 5% skimmed milk for 1 h. The specific proteins were detected with primary antibodies: β-actin (AC026), N-cadherin (A19083), E-cadherin (A3044), Vimentin (A11952), and Snail (A5243), which were purchased from ABclonal Technology Co. Ltd., China. The blots were thereafter treated with horseradish peroxidase-conjugated secondary antibodies. The bands were visualized using the ECL reagent and FluorChem M system (Protein Simple, Santa Clara, CA, USA).

### 4.12. Colony Formation

The cells (1 × 10^3^ cells/well) were seeded into a 6-well culture plate for one week, and miR-125b mimics were added into cells for 48 h. The colonies were fixed with 4% paraformaldehyde for 15 min and washed 3 times with PBS. Thereafter, crystal violet solution (0.1%) was added to wells and incubated for 10 min. After washing 3 times with PBS, the stained colonies were counted and photographed using a Nikon microscope. ImageJ software (National Institutes of Health) was used to quantify the number of colonies.

## 5. Conclusions

An association of miR-125b with miscarriage has been demonstrated previously. *VEGF* can serve as an important target gene of miR-125b that has been closely related to placental angiogenesis and trophoblast cell invasion [26]. This study also identified a possible relationship between *VEGF* and miscarriage. The findings indicated that miR-125b could significantly affect placental angiogenesis by inhibiting *VEGF* expression, and thus could possibly lead to the development of novel treatment modalities for PL.

## Figures and Tables

**Table 1:** List of primers used in the study.

Gene Name	Forward	Reverse
hsa-pri-miR-125b	CGGAATTCCTCGTGATCGTATGTGTATGTGCG	TTGCGGCCGCCATACACATAGCAGCC AACACGC
mmu-pri-miR-125b	CCGGAATTCTCCTTTGATACAACAGAC	TGCGGCCGCCTAACATACTATGCCTACTT
Chr9	CCGGAATTCCGGTCAAAGCATAAAC	TTGCGGCCGcCAGCAAACAGGTGGTC
U6	CTCGCTTCGGCAGCACA	AACGCTTCACGAATTTGCGT
miR-125b-5pRT	GTCGTATCCAGTGCGTGTCGTGGAGTCGGCAATTGCACTGGATACGACTCACAAG	
hsa-actin	AAGGAAGGCTGGAAGAGTGC	CTACAATGTGCTGCGTGTGG
mmu-actin	GATTCTGGCGATGGTGTAACTCA	AGATTCCATACCAATGAAAGAGGG

## Supplementary Materials

The following supporting information can be downloaded at: https://www.mdpi.com/article/10.3390/ijms23020943/s1.
