# Supplementary material for: Placenta-Specific miR-125b Overexpression Leads to Increased Rates of Pregnancy Loss in Mice
Source: Int J Mol Sci. 2022 Jan 15;23(2):943. doi: 10.3390/ijms23020943 (PMC8779150; doi:10.3390/ijms23020943)
Supplement: Supplementary file 1 [file ijms-23-00943-s001.zip › ijms-1496414-supplementary.pdf]

International Journal of
Molecular Sciences

MDPI

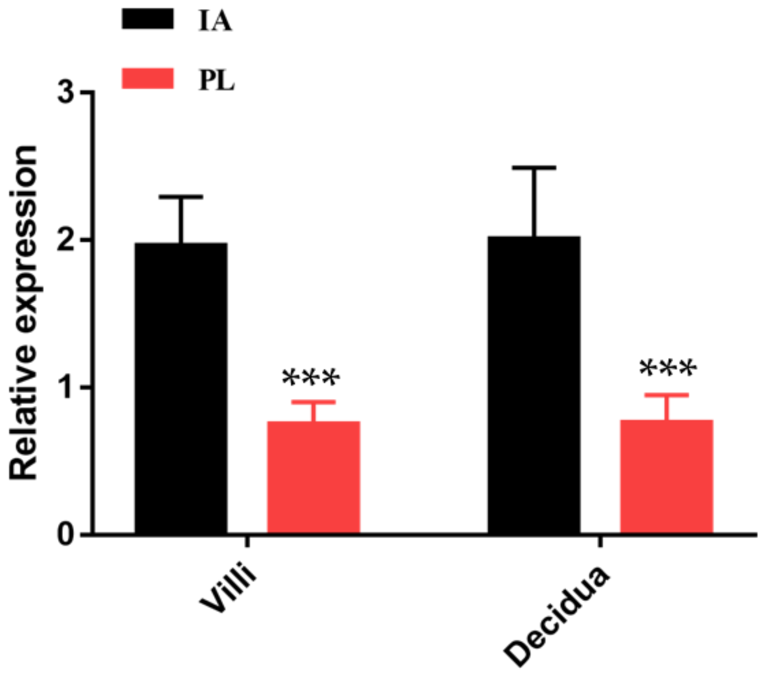

Figure S1. VEGF mRNA level in PL. An effective VEGF expression in PL placentas : The expression of VEGF in the decidua and villi of PL and IA. Data were presented as mean ± SEM, $n = 6$ (*** $p < 0.01$, vs. IA).

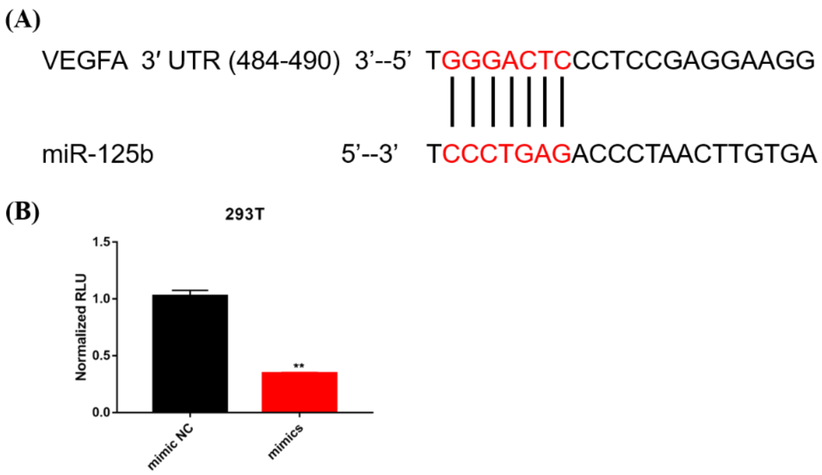

Figure S2. VEGFA may be a hypothetical direct miR-125b target (**A**) Luciferase report contains the miR-25b targeting sequences and mutated version of the VEGFA 3′ UTR wild type, including the site where the binding agent was altered (red). The luciferase reporter gene containing wild-type (Wt) VEGFA 3′ UTR (Mut) was cotransfected into 293t cells by simulating NC or miR-125b; (**B**) Luciferase activity was measured and normalized 72 h after transfection. Data were presented as mean ± SEM, $n= 3$ (** $p < 0.01$).

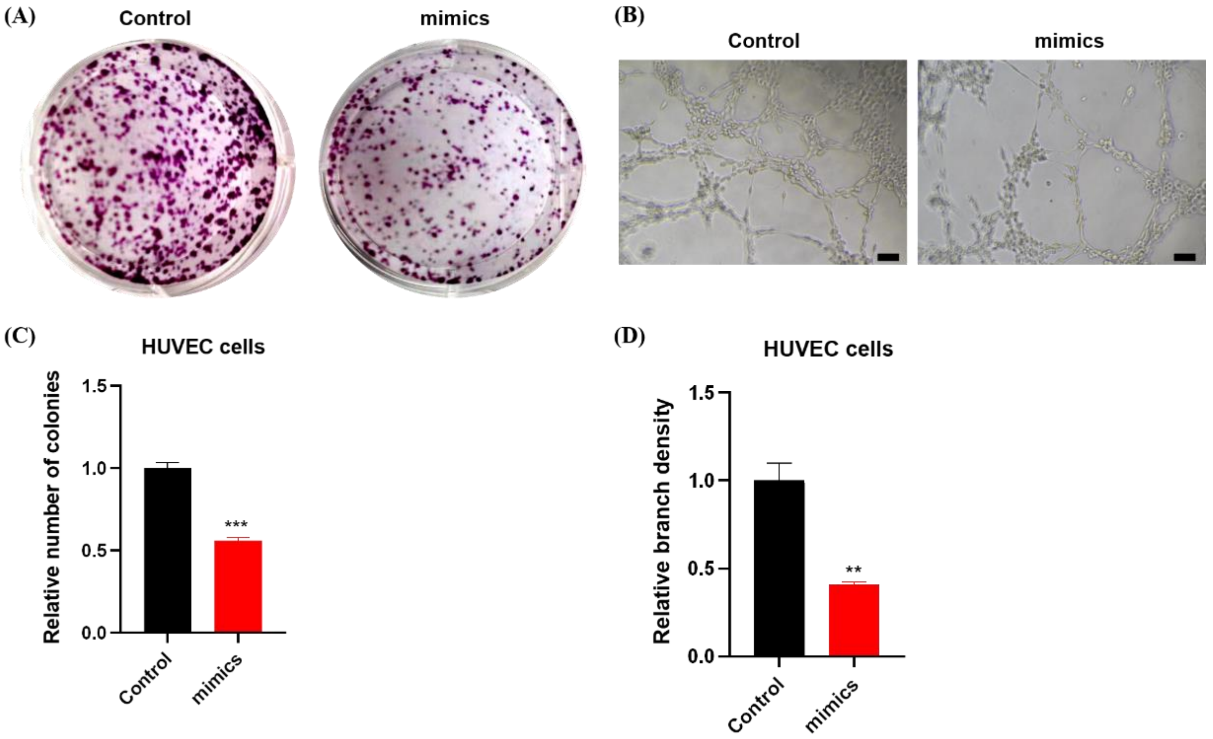

Figure S3. miR-125b inhibits angiogenesis. miR-125b inhibited the colony formation ability and angiogenesis of HUVEC cells (**A**,**C**) The result of the colony formation assays with HUVEC cells treated with miR-125b mimics. Data were presented as mean ± SEM, $n = 3$, ** $p < 0.01$ vs. control; (**B**) The pictures of tube formation. Scale bars: 200 μm; (**D**) The branch density of HUVEC after miR-125b mimics treatment. Data were presented as mean ± SEM, $n = 3$, ** $p < 0.01$, *** $p < 0.001$ vs. control.

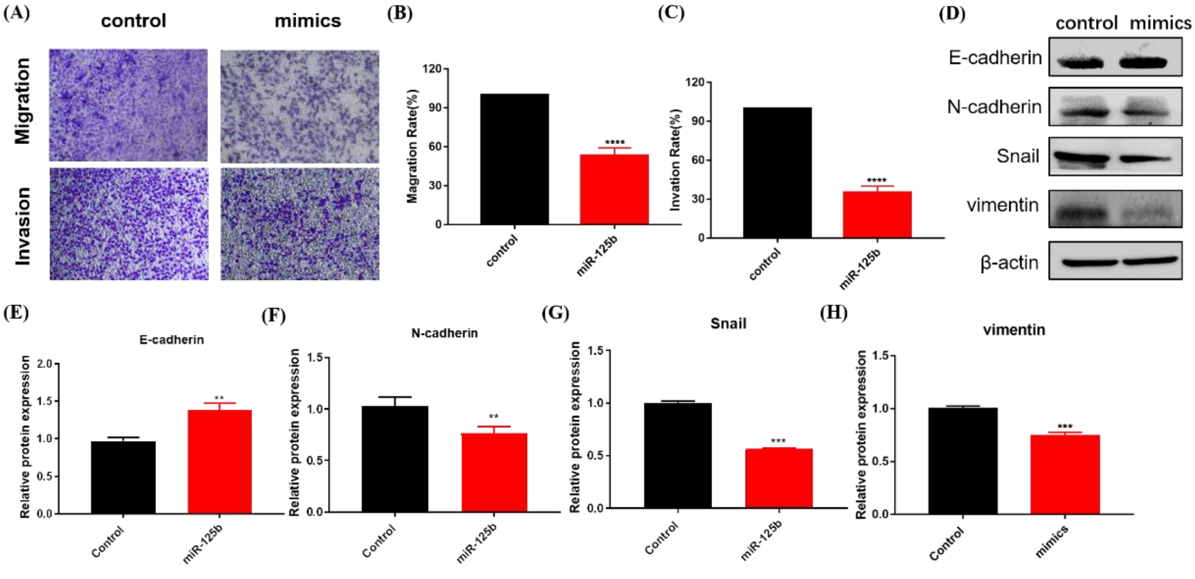

Figure S4. miR-125b suppressed the migration and invasion by inhibiting EMT (**A**) Transwell test was used to detect the effect of miR-125b on migration and invasion ability of HUVECs. (**B**) The invasion result of the Transwell assays with HUVECs treated with miR-125b mimics. (**C**) The migration result of the Transwell assays with HUVECs treated with miR-125b mimics. (**D**) Effects of miR-125b on the expression levels of the N-cadherin, E-cadherin, Vimentin, Snail determined by Western blot analysis. (E-H) The quantification of the N-cadherin, E-cadherin, Vimentin, Snail. Data were presented as mean ± SEM, $n = 3$, * $p < 0.05$, ** $p < 0.01$, **** $p < 0.001$ vs. control.
